# Neglected seed dispersers and research compartmentalisation: how much do we know about what we don't know?

**DOI:** 10.1111/nph.71294

**Published:** 2026-05-28

**Authors:** Sara Beatriz Mendes, Esther Sebastián‐González, Alistair G. Auffret, Irene Castañeda, Andy J. Green, Casper H. A. van Leeuwen, Christophe Baltzinger, Ádám Lovas‐Kiss, Isabel Donoso, Ricardo Soares, Ruben Heleno

**Affiliations:** ^1^ Department of Biodiversity, Macroecology and Biogeography, Faculty of Forest Sciences and Forest Ecology University of Göttingen Göttingen 37077 Germany; ^2^ Department of Ecology University of Alicante Alicante 03690 Spain; ^3^ ‘Ramón Margalef’ Multidisciplinary Institute for the study of the Environment (IMEM) University of Alicante Alicante 03690 Spain; ^4^ Department of Ecology Swedish University of Agricultural Sciences Box 7044 Uppsala 75007 Sweden; ^5^ Ecology and Genetics of Conservation and Restoration, UMR INRA 1202 BIOGECO Université de Bordeaux Pessac 33615 France; ^6^ Department of Conservation Biology and Global Change Estación Biológica de Doñana (EBD), CSIC Américo Vespucio 26 Seville 41092 Spain; ^7^ Department of Ecology, Radboud Institute for Biological and Environmental Sciences, Faculty of Science Radboud University Nijmegen Nijmegen AJ 6525 the Netherlands; ^8^ Research Unit Forest Ecosystems INRAE Val de Loire Nogent‐sur‐Vernisson 45290 France; ^9^ MTA‐HUN‐REN Centre for Ecological Research Momentum Dispersal Ecology Research Group Debrecen 4026 Hungary; ^10^ Department of Planetary Health, Faculty of Health Sciences, Institute of One Health University of Debrecen Debrecen 4032 Hungary; ^11^ BC3 Basque Centre for Climate Change Scientific Campus of the University of the Basque Country Sede Building 1, 1st floor Barrio Sarriena s/n Leioa 48940 Spain; ^12^ Ikerbasque Basque Foundation for Science Plaza Euskadi 5 Bilbao 48009 Spain; ^13^ Centre for Functional Ecology, Associate Laboratory TERRA, Department of Life Sciences University of Coimbra Calçada Martim de Freitas Coimbra 3000‐456 Portugal

**Keywords:** diaspore, ecosystem services, frugivory, myrmecochory, publication bias, research silos, sample coverage, zoochory

## Abstract

Seed dispersal is critical for long‐term ecosystem resilience. However, excessive compartmentalisation of research into particular disperser guilds (e.g. birds) hampers our understanding of their relative contributions to overall seed dispersal, risking erroneous conclusions and overlooking key information gaps. Here, we evaluate how information about over 11 000 interactions between 1902 plant and 455 animal species in Europe accumulated over 400 years, and estimate information completeness across disperser guilds, plant types, and dispersal mechanisms (endozoochory, epizoochory, synzoochory, and myrmecochory). Information regarding the plants dispersed by each group of animals ranges from 27% to 79% sample coverage, with birds of prey (27%), ants (28%), and rodents (31%) particularly understudied. Most research focussed on fleshy‐fruited plants rather than dry‐fruited plants, and most on internal dispersal as the mechanism (endozoochory). Interestingly, scientists' perceptions of what are the most neglected disperser guilds do not correlate with our sample coverage estimates, showing an inability of the scientific community to identify information gaps. We urge researchers to bridge research silos and to study overlooked guilds, mechanisms, and plant types. Implementing community‐level synthesis studies that integrate data across guilds is essential for accurately predicting ecosystem responses to global change and developing effective conservation strategies.

## Graphical abstract

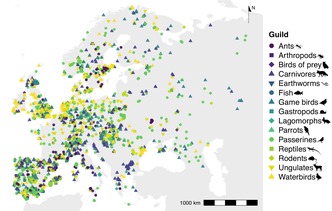

## Introduction

Global change drivers, such as anthropogenic climate change, habitat fragmentation, land‐use change, and biological invasions, are transforming ecosystems at an unprecedented rate (Maxwell *et al*., 2016; Beaugrand *et al*., 2019). In these dynamic environments, the long‐term persistence of plant and animal species depends on their capacity to colonise newly available habitats. However, this may be challenging for plants due to their sessile nature (Kokko & López‐Sepulcre, 2006; Chen *et al*., 2011). A large majority of all plant species rely on seed dispersal by animals (i.e. zoochory) for their ability to adjust to global changes (Aslan *et al*., 2013; Schleuning *et al*., 2016). This makes animal‐mediated dispersal a critical ecological function and a key regulating service that underlies long‐term vegetation dynamics (Traveset *et al*., 2014; IPBES, 2019), particularly by allowing plants to track their suitable climatic envelopes in a rapidly warming planet (Lovas‐Kiss *et al*., 2023; Mendes *et al*., 2025), enabling plant populations to recover after a disturbance (e.g. deforestation, wildfire, or floods) (Benedicto‐Royuela *et al*., 2023; Landim *et al*., 2026) and to colonise new habitats (Wasowicz *et al*., 2025).

Over 400 animal species, including arthropods, birds, mammals, and fish, are known to disperse seeds in Europe (Mendes *et al*., 2024). However, most seed dispersal research is highly compartmentalised on specific disperser guilds, because each disperser guild typically requires specific protocols, permits, and expertise. For example, many publications focus only on dispersal of fleshy‐fruited plants by birds (e.g. González‐Varo *et al*., 2021) or on dispersal by waterbirds (e.g. Green *et al*., 2023), ungulates (e.g. Albert *et al*., 2015), carnivorous mammals (e.g. González‐Varo *et al*., 2015), or ants (e.g. Retana *et al*., 2004). Similarly, most studies focus on seed dispersal by a single mechanism, usually either internal (endozoochory) (e.g. Cruz *et al*., 2013) or external dispersal (epizoochory) (e.g. Couvreur *et al*., 2004), or on only one fruit type, such as fleshy‐fruited plant species (e.g. Mendes *et al*., 2025). However, it is increasingly recognised that different seed dispersal mechanisms and vectors can be redundant or complementary to one another in terms of contributing to effective seed dispersal (Christianini & Oliveira, 2010; Costa *et al*., 2014; Albert *et al*., 2015) and that animals disperse many plants categorised into abiotic dispersal syndromes (González‐Varo *et al*., 2024; Wasowicz *et al*., 2025; van Leeuwen *et al*., 2026).

The compartmentalisation of seed dispersal research leads to research silos (i.e. the isolation of knowledge within distinct subfields; Editorial, 2016; Cunningham & Greene, 2025), which hinder comparisons of the contributions of different disperser guilds to seed dispersal, and hamper our ability to identify existing knowledge gaps from a broader taxonomic and functional perspective (Escribano‐Avila *et al*., 2018). Moreover, research silos can lead to each group of researchers perceiving their own field as neglected and continuing their research along the same lines, with little or no interaction across subfields of dispersal ecology. This may eventually lead to erroneous conclusions and misinformed conservation efforts for plants and animals (Ribeiro *et al*., 2016; Hughes *et al*., 2021).

Correctly identifying research gaps in seed dispersal (i.e. what we do not know) requires overcoming specific research silos and integrating data and ideas across subfields to correct likely biased perceptions of neglected research areas (i.e. how much do we know about what we do not know). Such information integration will lead to more comprehensive research questions, better‐informed hypotheses, and more robust methodological approaches, ultimately advancing the researchers' ability to address complex ecological challenges. Here, we leverage one of the most complete seed‐dispersal datasets to date, encompassing all known dispersal interactions for an entire continent (Mendes *et al*., 2024), to identify which disperser guilds, dispersal mechanisms, and fruit types are empirically best studied (in relation to their estimated number of interactions). Such an exercise allows us to estimate for which taxonomic groups future studies are most likely to yield the highest marginal additional return of new information. Finally, we compare these results with researchers' stated perceptions of which disperser guilds are understudied, thereby evaluating how closely these perceptions align with actual neglected guilds.

## What we know: a large body of slowly accruing information

Knowledge about which animal species disperse the seeds of each plant species in Europe began to accumulate slowly in the late 17^th^ century and has seen a huge acceleration in recent decades, reaching several dozen publications per year (Mendes *et al*., 2024). These data have recently been compiled into a large dataset that includes records of seed dispersal and frugivory by birds, mammals, reptiles, fish, ants, beetles, slugs, snails, and earthworms from nearly 2000 publications across Europe (Mendes *et al*., 2024).

**Fig. 1:**
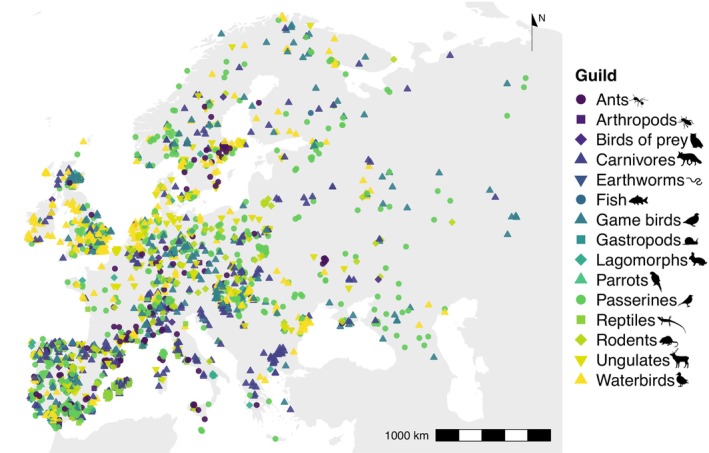
Geographic distribution of seed dispersal studies by disperser guild. Each point represents the location of a published source documenting seed dispersal interactions for the corresponding guild. Points are slightly jittered to reduce overlap. A more detailed version of this figure is provided in Supporting Information Fig. S1. Animal image credits: phylopic.org under CC0 1.0 universal licence (https://creativecommons.org/publicdomain/zero/1.0/).

We used the dataset of Mendes *et al.* (2024) to estimate the information completeness for different disperser guilds, dispersal mechanisms, and plant types regarding seed dispersal interactions in Europe. For this, we focussed exclusively on information related to likely seed dispersal (i.e. only when seeds or fruits were identified in the faeces, digestive tract, pellets, fur, feather, hooves, or nests, irrespective of whether or not seed viability was experimentally confirmed) and excluded records where seed fate was unknown (frugivory) or when seeds were destroyed (seed predation) (Heleno *et al*., 2011; Mendes *et al*., 2024). All records were resolved to the lowest possible taxonomic level, but only those records in which both interaction partners could be identified to the species level were retained for analysis. Overall, 22% of the interactions reported in the literature included supra‐specific taxa – likely reflecting researchers' focus on guilds in which they had greatest taxonomic expertise – and were therefore excluded (we kept 31 486 out of 40 152 records). Finally, this filtering resulted in a dataset comprising 11 413 interactions, between 1902 plant species and 455 animal species, compiled from 1412 publications across 37 countries (Fig. 1, Supporting Information Fig. S1).

Disperser species were classified into 15 commonly used seed dispersal guilds (Tables 1, S1), and plant species were categorised as bearing fleshy or dry fruits (Table 1). Furthermore, observed dispersal mechanisms were classified into endozoochory (internal dispersal), epizoochory (passive external dispersal), myrmecochory (active external dispersal by ants), or synzoochory (actively carried by vertebrates) (Table 1). While commonly used, these categories represent only an analytical simplification of the inherent complexity of seed dispersal interactions.

**Table 1:** Number of publications reporting interactions from each disperser guild, dispersal mechanism, and plant type, including information about the number of species included in the group, their sample coverage, the number of interactions observed in the group, and the asymptotic estimate of total pairwise interaction richness (Chao richness estimator, *q* = 0) computed using incidence‐based rarefaction and extrapolation with the inext package (Hsieh *et al.*, 2024).

		No. of publications	No. of species	Sample coverage	Observed interactions	No. of estimated interactions (mean ± SE)
Disperser guild	Carnivores	301	23	79%	672	1315 (±78)
	Lagomorphs	46	5	69%	237	342 (±23)
	Passerines	442	150	68%	3119	7064 (±167)
	Ungulates	131	16	65%	2740	6439 (±180)
	Reptiles	41	11	60%	86	135 (±20)
	Game birds	180	23	57%	1293	3428 (±144)
	Waterbirds	196	87	55%	1824	5043 (±178)
	Rodents	103	25	31%	334	1722 (±247)
	Ants	95	75	28%	947	5166 (±427)
	Birds of prey	15	14	27%	76	255 (±82)
	Fish	2	2		4	
	Earthworms	1	1		1	
	Arthropods (except ants)	9	10		24	
	Gastropods	5	4		6	
	Parrots	1	9		50	
Dispersal mechanism	Endozoochory	1156		64%	9602	23 190 (±335)
	Epizoochory	29		38%	879	2739 (±164)
	Synzoochory	169		36%	479	1860 (±175)
	Myrmecochory	95		28%	947	5166 (±369)
Plant type	Fleshy	906	276	73%	3367	6967 (±148)
	Dry	914	1626	55%	8046	22 501 (±365)

Sample coverage could not be reliably estimated for guilds with fewer than 15 publications.

Passerines were the most‐studied guild, appearing in 31% of the publications, followed by carnivores (21%) and waterbirds (14%) (Figs 1, S1; Table 1). By contrast, birds of prey, nonant arthropods, gastropods, fish, parrots, and earthworms received the least research attention, each accounting for *c*. 1% or less of the studies (Figs 1, S1; Table 1). As for the mechanisms by which seeds have been dispersed, the vast majority (82%) of studies have focussed on internal dispersal of seeds (endozoochory), followed by synzoochory (12%), myrmecochory (7%), and epizoochory (2%) (Table 1). Regarding fruit types, 65% of the studies included dry‐fruited species, and 64% included fleshy‐fruited species (some studies focussed on both fruit types; hence, the total exceeds 100%). Furthermore, only 12% of the studies experimentally confirmed the viability of the dispersed seeds through viability (e.g. tetrazolium) or germination experiments.

**Fig. 2:**
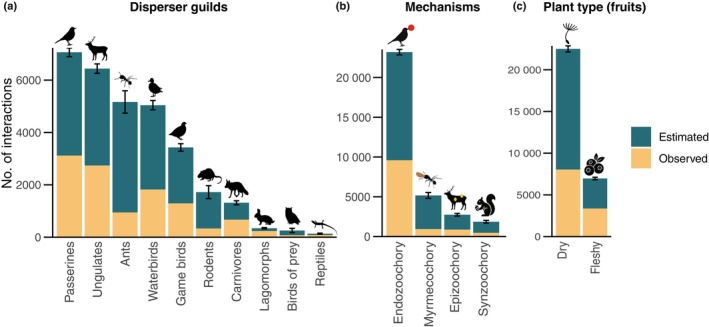
Comparison between the observed and estimated number of interactions for each (a) disperser guild, (b) dispersal mechanism, and (c) plant type (fruits). Only disperser guilds reported in 15 or more published sources, for which sample coverage could be robustly estimated, are shown. Orange bars represent the total number of observed interactions, while teal bars show the estimated number of interactions (mean ± SE). Animal image credits: phylopic.org under CC0 1.0 universal licence (https://creativecommons.org/publicdomain/zero/1.0/) and Illustrae.co.

Our findings reveal an uneven research focus among disperser guilds and dispersal mechanisms, which may limit our ability to assess the overall importance of dispersers for the European flora and may lead to incomplete estimates of the plant species dispersed by animals and to incomplete estimates of redundancy (e.g. multiple vectors of the same plant). Furthermore, this imbalance may compromise conservation and management decisions by causing the misidentification of the most important disperser guilds for the community or for target plant species. The disproportionate attention to endozoochory by birds and mammals may reflect their key role as seed dispersers in Europe (as these groups disperse most plant species) (Fig. 2) or could be driven by a combination of ease of access or study of certain disperser guilds, or by researchers staying within their research silos. Furthermore, a lack of funding opportunities to expand research focus, a shortage of taxonomists trained on invertebrate taxa (Caldwell *et al*., 2024), and the increasingly logistical challenges associated with documenting seed dispersal interactions by multiple dispersal guilds and mechanisms could also contribute to this imbalance. Therefore, effective progress in seed dispersal research requires integrating fragmented information across research silos (Shi & Evans, 2023) and accurately identifying those areas for which further information is particularly needed.

## What we don't know: neglected areas in European seed dispersal

We identified existing information gaps on seed dispersal research by estimating sample coverage, that is how well the existing literature depicts the true diversity of the seed dispersal community in Europe. We did so by implementing the rarefaction and extrapolation framework proposed by Chao *et al*. (2014), using the R‐package iNEXT with 100 replications (Hsieh *et al*., 2024; R Core Team, 2025). To do this, we built incidence matrices for each disperser guild, dispersal mechanism, and plant type (fleshy vs dry‐fruited). In each matrix, rows corresponded to unique pairwise interactions (i.e. plant species *i* dispersed by animal species *j*), and columns corresponded to bibliographic references reporting each interaction (average = 2; range 1–34 references). Disperser guilds reported in less than 15 bibliographic references were excluded from sample coverage estimation due to unreliable extrapolation at lower sample sizes.

Our analysis revealed an overall sample coverage ranging between 27% and 79% across all disperser guilds, dispersal mechanisms, and plant types (Fig. 2; Table 1; Fig. S2). Birds of prey (27% sample coverage), ants (28%), and rodents (31%) emerged as the most neglected guilds, independently of their importance for overall seed dispersal (Fig. 2). Carnivores (79%) were the best‐studied group, and our knowledge is likely already relatively comprehensive for them (Figs 2, S2; Table 1).

**Fig. 3:**
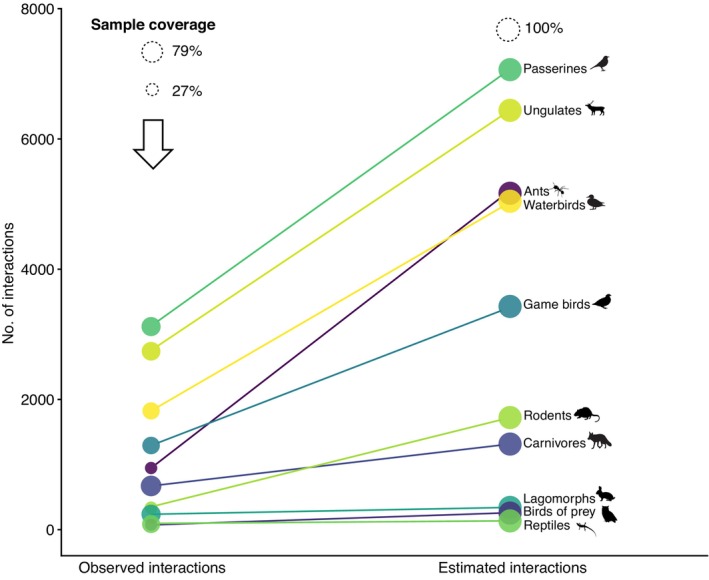
Observed and estimated number of seed dispersal interactions for each disperser guild, contrasting the order of ‘importance’ of each guild in terms of currently known vs estimated (mean) interactions. For each guild, the left circle (observed interactions) is sized proportionally to the sample coverage, and the right circle (estimated interactions) has a fixed size and represents the estimated total number of interactions (i.e. 100%). Lines connect each guild's observed and estimated values, highlighting changes in rank when estimated interactions are accounted for. Only disperser guilds reported in 15 or more published sources, for which sample coverage could be robustly estimated, are shown. Animal image credits: phylopic.org under CC0 1.0 universal licence (https://creativecommons.org/publicdomain/zero/1.0/).

Passerines (mean ± SE: 7064 ± 167 estimated interactions), ungulates (6439 ± 180), ants (5166 ± 427), and waterbirds (5043 ± 178) are the disperser guilds with the highest estimated interaction richness in Europe (Fig. 2; Table 1). Building on these findings, our results show that thousands of interactions remain to be discovered, not only among neglected guilds, such as ants (*c*. 4219 interactions still to discover) and rodents (*c*. 1388), but also among well‐studied guilds, such as passerines (*c*. 3945), ungulates (*c*. 3699), and waterbirds (*c*. 3219) (Fig. 3; Table 1). Moreover, most of the interactions yet to be discovered are not redundant (e.g. the first animal vectors identified for many dry‐fruited species). Notably, low sample coverage does not necessarily correspond to a large absolute information gap; for example, birds of prey, despite having the lowest coverage, have only *c*. 179 interactions remaining to be discovered (Fig. 3; Table 1). However, even guilds with lower estimated interaction richness deserve researchers' attention as they may play unique and key ecological roles in seed dispersal, such as facilitating rare long‐distance dispersal events or being the unique or main disperser of a plant species (e.g. Mendes *et al*., 2025).

Unfortunately, dispersal by nonant arthropods (nine studies), gastropods (five studies), fish (two studies), earthworms (one study), and parrots (one study) was too poorly represented to reliably estimate sample coverage. For these less‐investigated guilds, it is therefore still not possible to know whether they are poorly studied because they are relatively rare and with a limited role in seed dispersal, or whether they are neglected but potentially relevant seed dispersers in Europe. One exception is the parrots' guild, whose species have been recently introduced in Europe, which can explain the limited number of studies, while they are known to disperse seeds in their native range (e.g. Blanco *et al*., 2016). Finally, it is possible, and even likely, that some disperser guilds might not have been sampled at all (e.g. amphibians; da Silva *et al*., 1989).

As for the mechanisms by which seeds are dispersed, endozoochory is the one for which information is most complete (64% coverage), followed by epizoochory (38%) and synzoochory (36%). By contrast, seed dispersal by ants (myrmecochory) stands out as the least‐explored mechanism (only 28% coverage) (Figs 2, S2; Table 1). This implies that future studies on myrmecochory are most likely to provide a disproportionate amount of new information regarding seed dispersal interactions – and, notably, most of these interactions will involve seeds that lack a myrmecochorous syndrome (van Leeuwen *et al*., 2026). However, it is also important to note that, in absolute terms, more new interactions remain unobserved in already well‐sampled guilds (notably passerines and ungulates) than in other guilds.

Finally, we detected a considerably greater sample coverage for fleshy‐ than for dry‐fruited plant species (73% and 55%, respectively), reflecting a greater focus on the former. This could be explained by their disproportional attractiveness for animals and scientists, or by the difficulty in identifying seeds from dry‐fruited species. The lower estimated sample coverage for dry‐fruited plants contrasts with their much greater diversity in the European flora (Vargas *et al*., 2023) (Fig. 2). Future studies on dry fruit dispersal are therefore clearly needed (Wasowicz *et al*., 2025).

## How much do we know about what we don't know?

Our assessment of research coverage of different disperser guilds, mechanisms, and plant types identified information gaps in seed‐dispersal research. However, these gaps might not necessarily match the research community's perception of information gaps, which could misdirect future research efforts. We found strong evidence of research siloing, with 92% of the studies reporting interactions from a single guild of animal dispersers. Such dominance of guild‐centred studies hinders comparisons of the relative strength of the different disperser guilds involved in European seed dispersal.

**Fig. 4:**
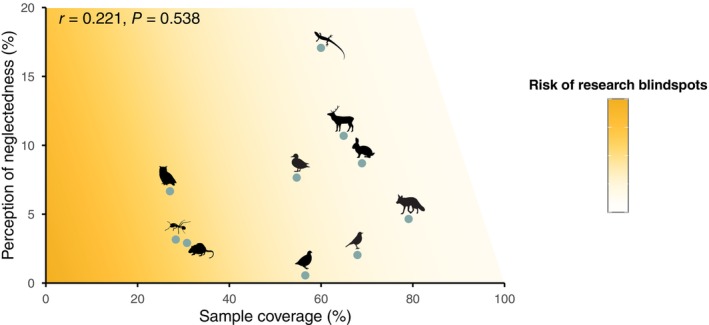
Correlation between sample coverage and perception of neglectedness of different dispersal guilds (estimated as the number of European seed dispersal studies (*n* = 388) claiming that a specific guild is neglected or understudied, in relation to the number of studies reporting interactions of that guild). The risk of research blind spots is higher for guilds with a low sample coverage but where that information deficit is not reflected in the perceptions of neglectedness by the scientific community. Guilds for which it was not possible to estimate sample coverage were excluded from this analysis. Animal image credit: phylopic.org under CC0 1.0 universal licence (https://creativecommons.org/publicdomain/zero/1.0/).

To test whether perceived research gaps (i.e. perceived understudied disperser guilds) match the identified ones (i.e. actually understudied disperser guilds), we extracted claims of neglected disperser guilds from the studies in the database that focussed primarily on seed dispersal (most publications only reported dispersal of seeds incidentally, often within the scope of diet studies; Mendes *et al*., 2024). This subset (*n* = 388 publications) has been identified by selecting all English‐language publications that use the terms: *chory, diaspor*, disper*, frugiv*, fruit, or seed* in the title, and then reading their abstracts. To identify the guilds considered neglected, we then searched the full texts for the terms ‘under*’, ‘neglect*’, ‘poor*’, ‘over*’, ‘stud*’, ‘few*’, ‘rar*’, ‘unrecogni*’, ‘ignor*’. Sixty‐six publications reported at least one overlooked guild, with a total of 12 animal disperser guilds reported as neglected. More importantly, we found that the stated perceptions of neglectedness (estimated as the number of studies claiming that a specific guild is neglected in relation to the number of studies reporting interactions of that guild) is not associated with the sample coverage of each guild (Pearson's correlation test, *n* = 10, *r* = 0.221, *P* = 0.538; Fig. 4). Such a mismatch reveals a worrying incapacity of the scientific community to identify neglected disperser guilds, likely because researchers' perceptions are shaped by the biases of their own research siloes.

Progress in seed dispersal research calls for multi‐guild studies at various spatial and temporal scales – including local studies (e.g. Eycott *et al*., 2007; Donatti *et al*., 2011; Jaroszewicz *et al*., 2013; Acosta‐Rojas *et al*., 2019). Nonetheless, pursuing such broad research goals can be particularly challenging due to funding constraints and the short‐term nature of most research programmes. Regular funding opportunities remain scarce, and multi‐guild research largely depends on occasional, large‐scale, competitive programmes (e.g. ERC, Biodiversa, National Science Foundation), compatible with taxonomically broad and temporarily sustained sampling designs sustained by large collaboration networks. This does not mean that there is no value in single‐guild or even species‐focussed studies. Indeed, such studies remain valuable *per se* and for providing data for future multi‐guild and synthesis studies.

The research effort dedicated to different disperser guilds may not be evenly distributed across the European continent (Figs 1, S1). If information from specific disperser guilds is spatially clustered, continental‐level estimates of sample coverage could be biased. To explore this possibility, we performed a sensitivity analysis to examine whether the relative sample coverage of each guild showed a consistent trend across various spatial resolutions. Thus, we divided the continent into 4 (2500 × 2500 km), 9 (1666 × 1666 km), 16 (1250 × 1250 km), 25 (1000 × 1000 km), or 36 (833 × 833 km) grid cells, representing increasingly finer spatial scales (Fig. S3), and calculated sample coverage for each disperser guild within these subdivisions. At finer resolutions, the dataset becomes too sparse to reliably estimate sample coverage due to the limited number of studies for each guild on each grid cell. However, overall trends in sample coverage remained largely consistent for each guild across these five levels of spatial aggregation (Fig. S4). Such consistency suggests that continental‐level estimates of sample coverage are likely informative and not biased by the spatial structure of the data.

## Conclusions

Our findings reveal that after four centuries of research reporting seeds dispersed by European animals, we might have recorded only about a third of all plant–animal pairwise interactions on the continent (observed/estimated interactions ≈37%). This estimate is, however, naturally limited by existing information and likely still overlooks animal species, mechanisms, habitats, or regions that have not yet been sufficiently explored. More importantly, the overwhelming dominance of studies focussing on a single guild of dispersers still prevents us from comparing the relative importance of different disperser guilds in Europe.

To attain a more complete view of the European seed dispersal network, research efforts should concentrate on neglected dispersers (notably ants, rodents, and birds of prey), on the dispersers of dry‐fruited species, and on the external dispersal of seeds. Furthermore, even well‐sampled guilds – such as passerines, ungulates, and waterbirds – harbour thousands of undiscovered interactions, many of them not redundant, indicating that substantial information gaps persist despite relatively high sampling effort. More importantly, future research must strive to implement multi‐guild and community‐level frameworks or conduct more syntheses that integrate information across guilds, mechanisms, and plant types. Without this data integration, our knowledge will always be compartmentalised. Ultimately, achieving these goals depends on funding agencies' willingness to support seed dispersal research.

Given the importance of the seed dispersal service to maintain and restore ecosystems in a rapidly changing world (Benedicto‐Royuela *et al*., 2023; Mendes *et al*., 2025), we emphasise the urgent need for crossing research siloes and strengthening collaboration networks to attain a more holistic and informative view of seed dispersal interactions.

## Competing interests

None declared.

## Author contributions

SBM, RH, ES‐G, AGA, IC, AJG, CL, CB, AL‐K and ID conceived the idea. SBM, RS and RH collected the data. SBM analysed the data. SBM and RH led the writing with contributions from ES‐G, AGA, IC, AJG, CL, CB, AL‐K and ID.

## Disclaimer

The New Phytologist Foundation remains neutral with regard to jurisdictional claims in maps and in any institutional affiliations.

## Supporting information


**Fig. S1** Geographic distribution of seed dispersal studies by disperser guild.
**Fig. S2** Interaction accumulation curves.
**Fig. S3** Spatial distribution of the data and different grid cell sizes used to test sample coverage estimates.
**Fig. S4** Variation of estimated sample coverage across increasing resolution of cell grid size.
**Table S1** Taxonomic composition of the seed dispersal guilds considered in this study.
Please note: Wiley is not responsible for the content or functionality of any Supporting Information supplied by the authors. Any queries (other than missing material) should be directed to the *New Phytologist* Central Office.

## Data Availability

The data that support the findings of this study are openly available in the Supporting Information (Figs S1–S3; Table S1) of this article and in figshare at doi: 10.6084/m9.figshare.31057939.
