# Supplementary material for: Neglected seed dispersers and research compartmentalisation: how much do we know about what we don't know?
Source: New Phytol. 2026 May 28;251(3):967–74. doi: 10.1111/nph.71294 (PMC13326527; doi:10.1111/nph.71294)
Supplement: Supplementary file 1 — Fig. S1 Geographic distribution of seed dispersal studies by disperser guild. Fig. S2 Interaction accumulation curves. Fig. S3 Spatial distribution of the data and different grid cell sizes used to test sample coverage estimates. Fig. S4 Variation of estimated sample coverage across increasing resolution of cell grid size. Table S1 Taxonomic composition of the seed dispersal guilds considered in this study. Please note: Wiley is not responsible for the content or functionality of any Supporting Information supplied by the authors. Any queries (other than missing material) should be directed to the New Phytologist Central Office. [file NPH-251-967-s001.docx]

## *New Phytologist* Supporting Information

Article title: **Neglected seed dispersers and research compartmentalisation: how much do we know about what we don’t know?**

Authors: Sara Beatriz Mendes; Esther Sebastián-González; Alistair G. Auffret; Irene Castañeda; Andy J. Green; Casper H.A. van Leeuwen; Christophe Baltzinger; Ádám Lovas-Kiss; Isabel Donoso; Ricardo Soares; Ruben Heleno

Article acceptance date: 6 May 2025

The following Supporting Information is available for this article:


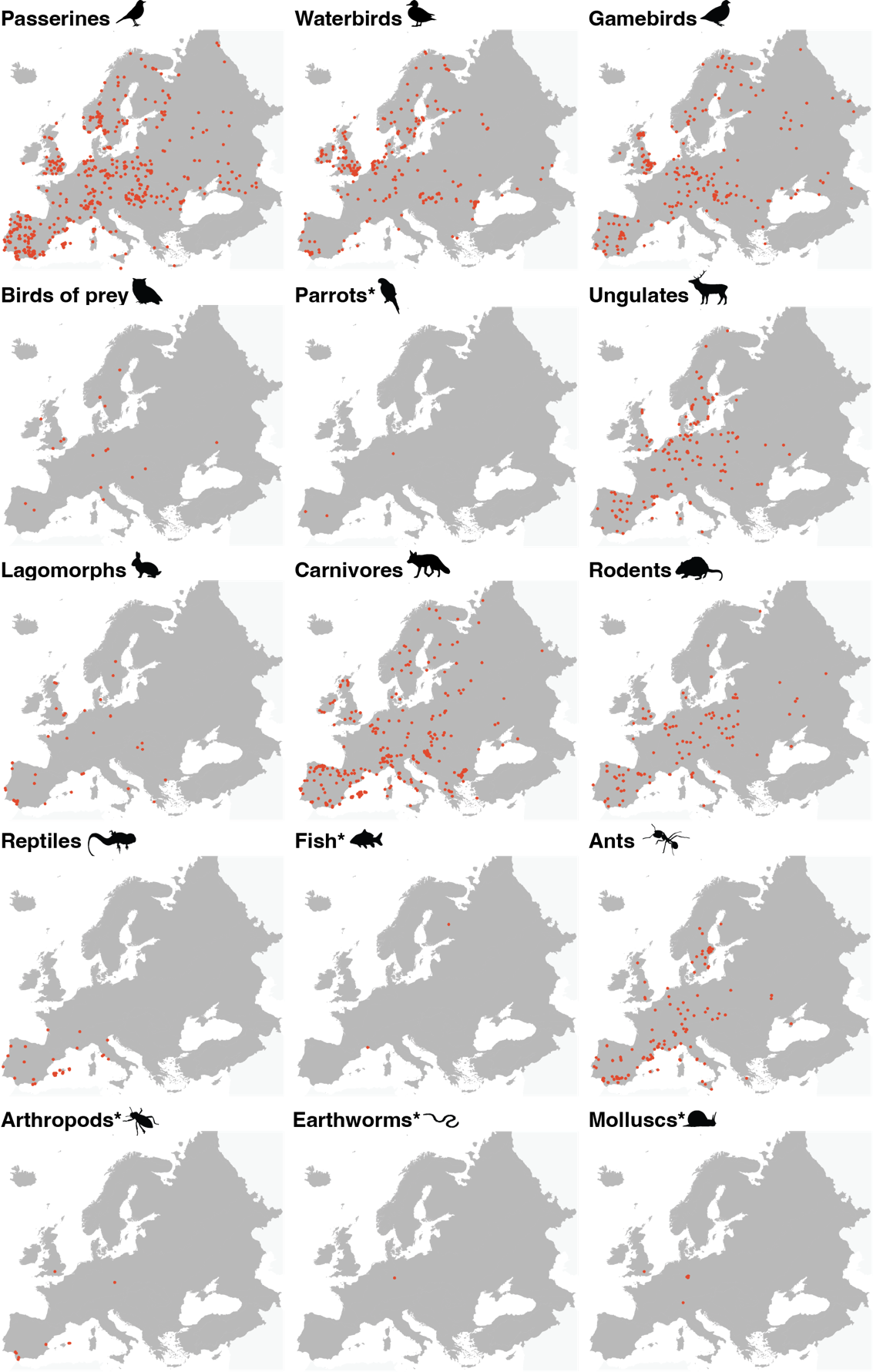


**Fig. S1. Geographic distribution of seed dispersal studies by disperser guild.** Each point represents the location of a published reference documenting seed dispersal interactions for the corresponding guild. Guilds with an asterisk have been excluded from the sample coverage estimate due to the reduced number of studies (Table 1). Animal image credit: phylopic.org under CC0 1.0.

#
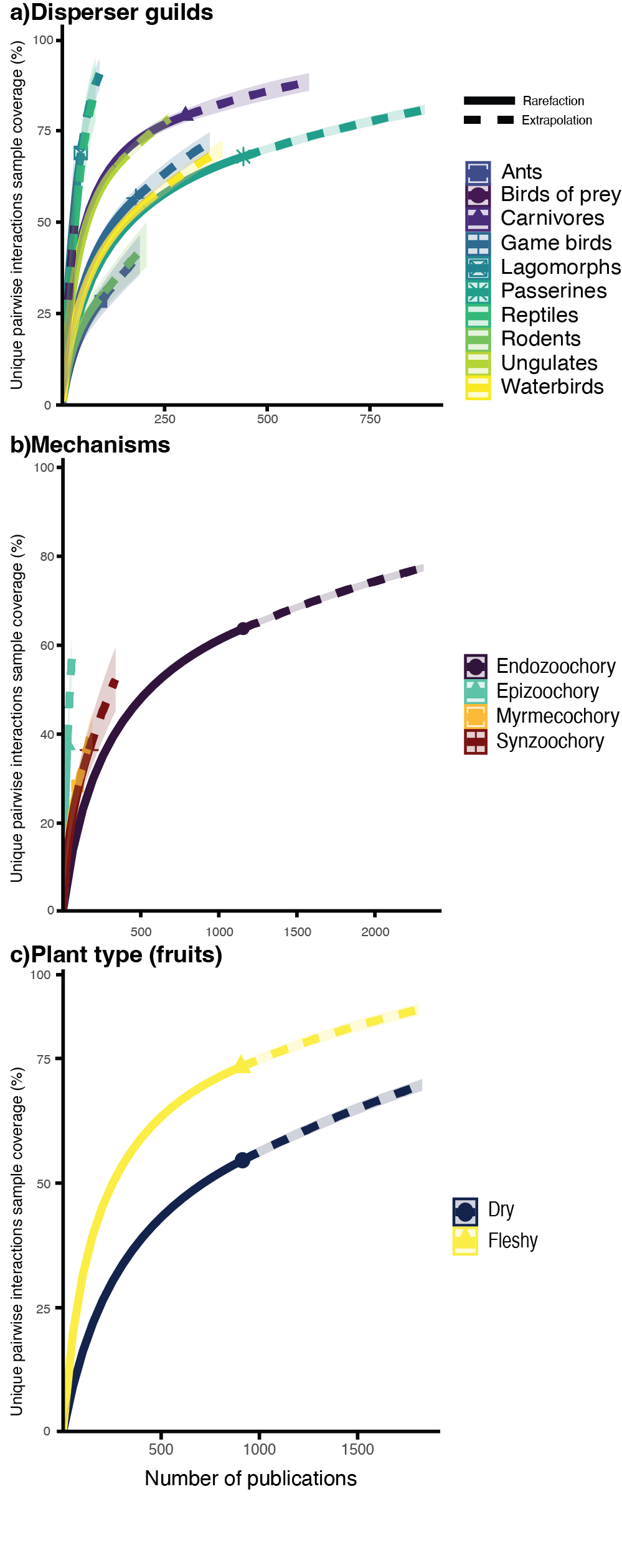


**Fig. S2.** Interaction accumulation curves for each a) disperser guild, b) dispersal mechanism and c) plant type. Estimates of sample coverage have been calculated with the R package iNEXT (Hsieh *et al.*, 2024; R Core Team, 2025).

**
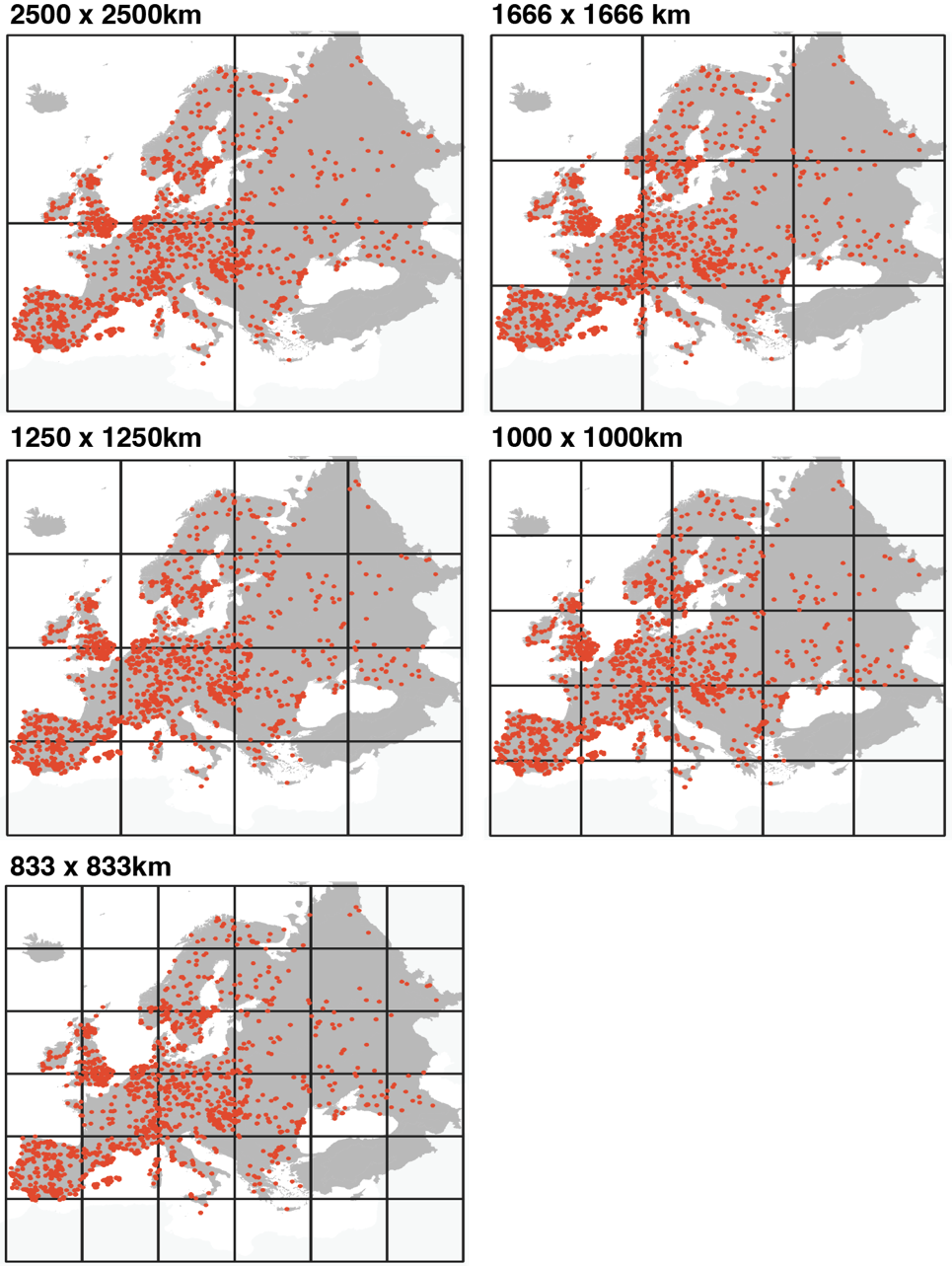
**

**Fig. S3.** Overall spatial distribution of the data used in this study and different grid cell sizes used to test the influence of spatial data distribution on sample coverage estimates (see Fig. S4).

**
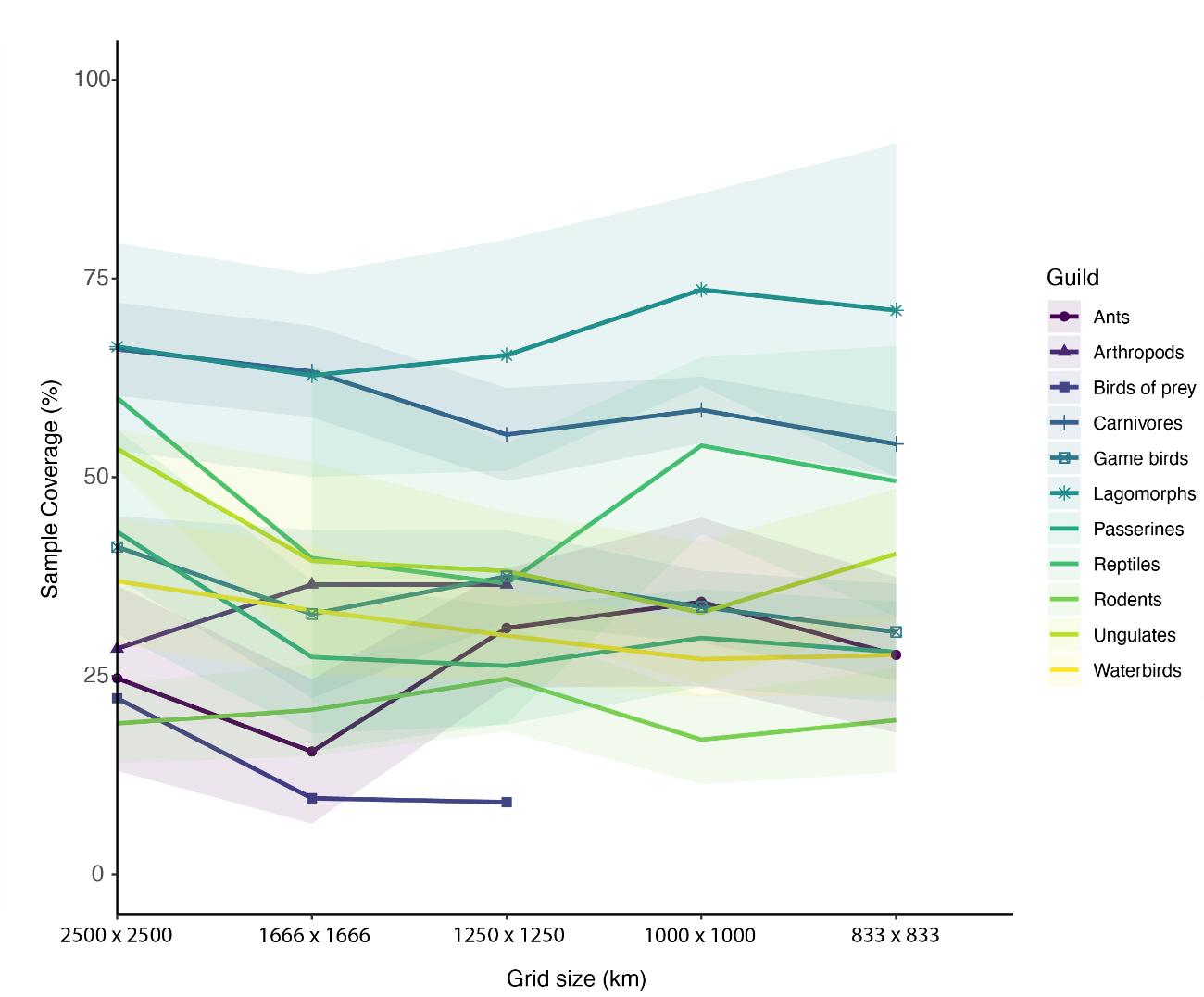
**

**Fig. S4.** Variation of estimated sample coverage across increasing resolution of cell grid size. No general pattern is detected, suggesting that any spatial aggregation of the data is not significantly biasing sample coverage estimates.

**Table S1.** Taxonomic composition of the seed dispersal guilds considered in this study.

| **Clade**  (Phylum or Class) | **Taxa included**  (Phylum, Class, Order, or Family) | **Guilds**  (this study) |
| --- | --- | --- |
| Annelida | Annelida | Earthworms |
| Arthropoda | Arthropoda (except Formicidae) | Arthropods |
|  | Formicidae | Ants |
| Actinopterygii | Actinopterygii | Fish |
| Aves | Falconiformes, Accipitriformes, Strigiformes | Birds of prey |
|  | Anseriformes, Charadriiformes, Ciconiiformes, Gruiformes, Pelecaniformes, Phoenicopteriformes, Podicipediformes, Suliformes | Waterbirds |
|  | Passeriformes, Bucerotiformes, Cuculiformes, Piciformes | Passerines |
|  | Columbiformes, Caprimulgiformes, Galliformes, Pterocliformes, Casuariiformes, Otidiformes | Game birds |
|  | Psittaciformes | Parrots |
| Mammalia | Artiodactyla, Perissodactyla | Ungulates |
|  | Carnivora, Erinaceomorpha, Soricidae, Cingulata | Carnivores |
|  | Lagomorpha | Lagomorphs |
|  | Rodentia | Rodents |
| Reptilia | Reptilia | Reptiles |
| Mollusca | Gastropoda | Gastropods |
